# Clinical analysis of infectious endophthalmitis following glaucoma filtration surgery

**DOI:** 10.1186/s12348-024-00391-4

**Published:** 2024-02-29

**Authors:** Liangliang Niu, Yan Luo, Huan Xu, Haili Huang, Rui Jiang, Xinghuai Sun

**Affiliations:** 1 Department of Ophthalmology & Visual Science, Eye Institute, Eye & ENT Hospital, Shanghai Medical College, Fudan University, Shanghai, 200031 China; 2 Hospital-Acquired Infection Control Department, Eye & ENT Hospital, Shanghai Medical College, Fudan University, Shanghai, 200031 China; 3 Ocular Trauma Center, Eye & ENT Hospital, Shanghai Medical College, Fudan University, Shanghai, 200031 China; 4 NHC Key Laboratory of Myopia, Chinese Academy of Medical Sciences, and Shanghai Key Laboratory of Visual Impairment and Restoration, Fudan University, Shanghai, 200031 China; 5 State Key Laboratory of Medical Neurobiology and MOE Frontiers Center for Brain Science, Institutes of Brain Science, Fudan University, Shanghai, 200032 China

**Keywords:** Bleb, Endophthalmitis, Glaucoma, COVID-19 pandemic

## Abstract

**Background:**

This study aimed to evaluate the clinical correlative factors and outcomes of treatment of bleb-associated endophthalmitis (BAE) following glaucoma filtration surgery in a Chinese population from the year 2012 to 2022, and to compare them with the clinical course during the coronavirus disease (COVID-19) pandemic period.

**Methods:**

This was a retrospective analysis of consecutive cases of BAE treated at the Eye & ENT Hospital of Fudan University, Shanghai, China, between January 1, 2012, and December 31, 2022. The clinical presentation, treatment modality, microbiological data, clinical course, and outcomes of visual acuity (VA) and intraocular pressure (IOP) in all BAE cases were collected and analyzed.

**Results:**

A total of 28 eyes with BAE were examined, predominantly in male patients (71.4%, *p* = 0.023). Most patients underwent trabeculectomy (89.3%, *p* ≤ 0.001), while a smaller proportion underwent Ex-PRESS implantation (10.7%). Primary open-angle glaucoma (POAG) was the most common type of glaucoma (39.3%, *p* ≤ 0.001). Most patients (96.4%) presented with poor visual acuity, worse than 20/400, and IOP ranged from 3–60 mmHg. Treatment, including initial tap-and-inject procedure of antibiotics (Ceftazidime and Norvancomycin) or initial pars plana vitrectomy (PPV), was initiated 5.0 ± 7.1 days after BAE onset. *Streptococcus* was the most common causative organism (53.6% of cases, *p* ≤ 0.001). The visual acuity significantly improved from 2.58 ± 0.27 to 2.14 ± 0.85 (reported in logMAR) after treatment (*p* ≤ 0.001), and most patients maintained normal tension during follow-up. Poisson regression model analysis showed the annual incidence of BAE during the COVID-19 pandemic period was significantly twice greater than that of previous years.

**Conclusions:**

BAE may cause irreversible visual impairment. POAG filtering surgery with male sex and the COVID-19 pandemic period might be potentially relevant factors for BAE. Culture positivity was closely related to BAE prognosis, with *Streptococcus* species being the leading pathogenic organisms. Online outpatient services, early diagnosis, and timely treatment may rescue vision and maintain IOP control in the presence of BAE.

## Background

Glaucoma is one of the leading causes of irreversible blindness worldwide. Glaucoma filtration surgery remains a classical treatment for adequately controlling intraocular pressure (IOP) [1, 2]. With the rapid development of medical technologies, glaucoma filtration surgeries are not limited to standard trabeculectomy. Glaucoma drainage implants, such as Ex-PRESS and Ahmed glaucoma valve implantation, have been used as alternative surgical approaches to trabeculectomy for glaucoma management [2–4].

In the presence of a filtering bleb, glaucoma filtration surgery carries the risk of infection in patients [5–7]. One of the most serious complications is bleb-associated endophthalmitis (BAE), which involves a combination of filtering bleb infection and vitritis [8, 9].

Although advances in surgical techniques have resulted in increased success rates following glaucoma filtering surgery [2, 3, 10], cases of patients developing BAE after the surgery have been reported, resulting in poor and irreversible visual impairment, occasionally leading to phthisis or enucleation [8, 9, 11]. In particular, during the pandemic, people have faced enormous challenges in medical treatment throughout China over the past 3 years. Therefore, we conducted a comprehensive analysis of the clinical manifestations, microbial spectrum, treatment, and prognosis of bleb-associated endophthalmitis from 2012–2022 at our institution, a large ophthalmology center in China, and compared the clinical course during the coronavirus disease-19 (COVID-19) pandemic and previous years.

## Methods

A retrospective study was conducted on all patients with BAE who presented to the Eye & ENT Hospital of Fudan University, Shanghai, between January 1, 2012, and December 31, 2022. The Ethics Committee approved the study.

Using a clinical case database, patients diagnosed with endophthalmitis associated with previous glaucoma filtration surgery were preliminary screened for this study. Then patients with bleb infections and vitreous involvement were included in accordance with the diagnosis of BAE [11, 12].

The patients’ demographics, type of glaucoma, previous glaucoma filtration surgical characteristics, location of blebs, time from the procedure to endophthalmitis, presenting complaints, slit lamp examination findings, time from symptoms to treatment, treatment regimen, and organism culture results were examined. The Seidel test was applied to observe the bleb condition. IOP (measured by Canon, TX-20) at the onset of BAE and final follow-up was recorded. All visual acuities during the study course are reported in Snellen and logMAR.

To detect the causative infectious agents, specimens were acquired from the aqueous and vitreous chambers. Standard microbiological detection protocols were applied to the speciation of both Gram- and Giemsa-stained bacteria.

Although most patients underwent glaucoma filtration surgeries at other primary hospitals, the therapies for BAE were all conducted at the Eye & ENT Hospital, which is one of the largest ophthalmology centers. The therapeutic regimen included an initial tap-and-inject procedure (injection of intravitreal antibiotics (such as Ceftazidime and Norvancomycin first), followed by pars plana vitrectomy (PPV) and bleb manipulation, or initial PPV (vitrectomy as first-line treatment) along with bleb manipulation. Successful control of inflammation and maintenance of the eyeball (neither atrophy nor enucleation at the final follow-up) were indicators of effective treatment.

All data were analyzed using R statistical software (R Core Team, Version 3.6.1). Normally distributed measurement data were represented as mean ± standard deviation (SD), and comparisons between groups were analyzed using the Student’s t-test. The chi-square test was used for comparisons between the two groups. Fisher’s exact probability test was used if the theoretical number of variables was < 10. Poisson regression model was used to estimate annual case numbers with a particular emphasis on the impact of the COVID-19 pandemic from 2020–2022. An offset was incorporated into the analysis to accommodate temporal variations. A *p*-value < 0.05 was considered significant.

## Results

### Patient demographics

A total of 28 eyes from 28 patients with BAE were presented at the Eye & ENT Hospital between 2012–2022. The patient’s demographic characteristics are listed in Table 1. A total of 20 (71.4%, *p* = 0.023) eyes were from male patients, and all were Mongoloid Chinese located in East China. The mean age at the time of presentation was 49.5 ± 13.8 years, with a range of 24–74 years. Systemic diseases included hypertension in five (17.9%) patients, diabetes mellitus in two (7.1%) patients, coronary heart disease in two (7.1%) patients, and a history of cerebral infarction in one patient (3.6%). None of the patients had received anticancer or immunosuppressive therapy.

**Table 1:** Baseline demographic for the 28 patients with BAE

Characteristics	Values	***P***
Age, y		
Mean ± SD	49.5 ± 13.8	
Range	24–74	
Gender, n (%)		0.023
Female	8 (28.6%)	
Male	20 (71.4%)	
Race		
Mongoloid Chinese, n (%)	28 (100%)	
Systemic diseases, n (%)		0.308
Hypertension	5 (17.9%)	
Diabetes mellitus	2 (7.1%)	
Coronary heart disease	2 (7.1%)	
History of cerebral infarction	1 (3.6%)	
Without systemic diseases	18 (64.3%)	
Glaucoma type, n (%)		≤ 0.001
Primary open-angle glaucoma	11 (39.3%)	
Primary angle closure glaucoma	6 (21.4%)	
Primary congenital glaucoma	2 (7.1%)	
Possner-Schlossman syndrome	2 (7.1%)	
Traumatic glaucoma	1 (3.6%)	
Sturge-Weber syndrome	1 (3.6%)	
Corticosteroid glaucoma	2 (7.1%)	
Uveitic glaucoma	1 (3.6%)	
Unknown	2 (7.1%)	
Previous glaucoma filtration surgery, n (%)		≤ 0.001
Trabeculectomy	25 (89.3%)	
Ex-PRESS implantation	3 (10.7%)	
Bleb location, n (%)		
Superior	28 (100%)	
Adjunctive antimetabolite used, n (%)	Unknown^a^	

*BAE* Bleb-associated endophthalmitis

^a^It was noticed that nine patients who underwent the surgery in our hospital were underwent adjunctive antimetabolite mitomycin-C (MMC, 0.4 mg/ml) for 5 min during the surgical procedure

Among the eyes examined, 11 eyes (39.3%) had primary open-angle glaucoma (POAG), 6 (21.4%) eyes had primary angle closure glaucoma (PACG), 2 (7.1%) eyes had primary congenital glaucoma, 2 (7.1%) eyes had Possner-Schlossman syndrome, 1 (3.6%) eye had Sturge-Weber syndrome, 2 (7.1%) eyes had corticosteroid glaucoma because of the usage of corticosteroid eye drops, 1 (3.6%) eye had traumatic glaucoma, and 1 (3.6%) eye had uveitic glaucoma. In addition, two (7.1%) eyes were unknown on the glaucoma classification. There was significant difference between the glaucoma types and POAG was the most common type of glaucoma (39.3%, *p* ≤ 0.001).

Among the patients with BAE, 25 (89.3%, *p* ≤ 0.001) eyes underwent trabeculectomy, and 3 (10.7%) eyes underwent Ex-PRESS implantation. All blebs formation were located superiorly. As most patients (19 patients, 65.5%) underwent glaucoma filtration surgery in other primary hospitals previously, gathering statistics on the proportion of the adjunctive antimetabolites was difficult. It was noticed that nine patients who underwent the surgery in our hospital were underwent adjunctive antimetabolite mitomycin-C (MMC, 0.4 mg/ml, Zhejiang Hisun Pharmaceutical Co., LTD) for 5 min during the surgical procedure.

With the aid of a clinical case database, we listed the total number of patients who underwent glaucoma filtration surgery in our hospital and calculated the incidence of BAE from the year 2012 to 2022. We found that approximately 9161 patients underwent glaucoma filtration surgery in our hospital in the last decade, and the proportion of BAE was approximately 0.3%.

### Clinical characteristics

The cases (100%, *n* = 28) in our study were classified as delayed-onset (> 6 weeks following the glaucoma filtration surgery) endophthalmitis [12]. The interval between the surgery and the onset of BAE was 9.8 ± 5.4 years, ranging from 2 months to 20 years (Table 2). Most of the patients visited the outpatient or emergency departments with complaints of redness (86.2%), reduction of vision (82.1%), or ocular pain (71.4%). In most cases, conjunctival congestion (100%), non-visible fundus (100%), corneal edema (86.2%), and hypopyon (71.4%) on slit lamp examination were documented. The features of blebs at the time of BAE definition are summarized in Table 2. The Seidel testing demonstrated bleb leaks in nine (32.2%) cases. Pus in blebs and thin blebs occurred in 46.8% and 42.9% of cases, respectively. Five (17.9%) eyes presented with avascular blebs.

**Table 2:** Characteristics of presentation for the 28 patients with BAE

**Characteristics**	**Values**
Interval between glaucoma surgery and onset of BAE	
Mean ± SD, y	9.8 ± 5.4
Range	2 months–20 years
Presenting complaints, n (%)	
Redness	25 (86.2%)
Reduction of vision	23 (82.1%)
Ocular pain	20 (71.4%)
Increased eye secretion	5 (17.9%)
Increased lacrimation	5 (17.9%)
Foreign body sensation	1 (3.6%)
Slit lamp examination findings, n (%)	
Conjunctival congestion	28 (100%)
Corneal edema	25 (86.2%)
Hypopyon	20 (71.4%)
Bleb leak	9 (32.2%)
Avascular bleb	5 (17.9%)
Pus in bleb	13 (46.8%)
Thin bleb	12 (42.9%)
Non-visible fundus	28 (100%)
Interval between onset of symptoms and treatment	
Mean ± SD, d	5.0 ± 7.18
Range	10 h–30 days

*BAE* Bleb-associated endophthalmitis

The presenting VA was poor in most individuals, with hand movement (HM) and no light perception (NLP) at the onset of endophthalmitis. Only two cases (7.1%) had counting fingers or better visual acuity (Table 5). The mean IOP at the time of diagnosis was 18.6 ± 13.8 mmHg, ranging from 3–60 mmHg. Among them, five (17.9%) eyes exhibited abnormally high intraocular pressure of > 30 mmHg, and one (3.6%) case exhibited hypotony of less than 6 mmHg (Table 5).

### Management

The therapies administered to the patients with BAE are shown in Table 3. All patients underwent surgery at the Eye and ENT Hospital when they were diagnosed with endophthalmitis. Among those, one patient underwent PPV twice in another hospital and was transferred to our hospital for the third PPV. The interval between the onset of symptoms and treatment was 5.0 ± 7.1 days (interquartile range: 10 h–30 days). A total of 12 (42.9%) patients who came to visit the emergency room at midnight underwent an initial tap-and-inject procedure, followed by PPV and bleb manipulation the next day. A total of 16 (57.1%) patients underwent an initial PPV and bleb manipulation directly on the day of admission. All patients were administered ceftazidime and norvancomycin intraocularly. Two patients (7.1%) received steroid intravitreal injections, and two patients (7.1%) were administered antifungal agent intravitreal injections based on clinical judgment.

**Table 3:** Management of the 28 patients with BAE

**Treatment**	**Values (n, %)**	***P***
PPV combined with tap-and-inject	28 (100%)	0.423
Initial tap-and-inject	12 (42.9%)	
Initial PPV	16 (57.1%)	
Number of vitrectomy		< 0.001
1	24 (85.7%)	
2	3 (10.7%)	
3	1 (3.6%)	
Vitrectomy with Silicone Oil tamponade	6 (21.4%)	
Intravitreal medical therapy		
Ceftazidime	28 (100%)	
Norvancomycin	28 (100%)	
Steroids	2 (7.1%)	
Antifungal agents	2 (7.1%)	

*PPV* Pars plana vitrectomy, *BAE* Bleb-associated endophthalmitis

Twenty-four patients (85.7%) received single PPV surgery. Three patients (10.7%) received secondary PPV and one patient (3.6%) received third PPV (twice in another hospital and once in our hospital) as a result of excessive vitreous inflammation. Among these, 85.7% of patients received single PPV, which was the highest percentage in BAE patients’ PPV treatment (*p* ≤ 0.001). Six eyes (21.4%) were received vitrectomy with silicone oil tamponade.

### Microbiologic culture

Microbiologic culture results are available for 15 of 28 patients (63.6%), as shown in Table 4. Among culture-positive cases, 13 patients (46.4%) had Gram-positive bacteria in the aqueous or vitreous chamber. The most common causative organism was *Streptococcus spp.,* which was detected in 5 cases (17.9%) among culture-positive cases. Organisms discovered Staphylococcus in four patients (14.3%). However, specimens from the other four patients (14.3%) only indicated gram-positive bacteria in the bacterial smear process. Moreover, *Psedomonas stutzeri*, a gram-negative bacterium, was isolated from one patient (3.6%). *Candida albicans*, a type of fungi, was identified in another patient (3.6%). Significant difference between organisms were observed in Gram-positive bacteria, Gram-negative bacteria, and fungi culture results, and Gram-positive bacteria were the most common organisms in the 28 patients with BAE(*p* ≤ 0.001).

**Table 4:** Organisms culture results for the 28 patients with BAE

**Organism**	**No. of isolates (%)**
Numbers of positive culture	15 (53.6%)
Gram-positive bacteria	13 (46.4%)
Streptococcus viridans	3 (10.7%)
Streptococcus mutans	1 (3.6%)
Streptococcus pneumonia	1 (3.6%)
Staphylococcus epidermidis	1 (3.6%)
Staphylococcus sciuri	1 (3.6%)
Staphylococcus hominis	1 (3.6%)
Coagulase-negative staphylococcus	1 (3.6%)
Gram-positive bacteria only detected by bacterial smear	4 (14.3%)
Gram-negative bacteria	1 (3.6%)
Psedomonas stutzeri	1 (3.6%)
Fungi	1 (3.6%)
Candida albicans	1 (3.6%)
P*	≤ 0.001

*BAE* Bleb-associated endophthalmitis

P* Significant difference between organisms were observed in Gram-positive bacteria, Gram-negative bacteria, and fungi culture results, and Gram-positive bacteria were the most common organisms in the 28 patients with BAE(*p* ≤ 0.001)

### Follow-up results

The median follow-up duration was 3.3 ± 3.3 years (interquartile range 1 month-11 years) after hospital discharge. To better illustrate the follow-up results, the VA and IOP values from the time of BAE diagnosis to the final follow-up are listed in Table 5.

**Table 5:** Follow-up results for the 28 patients with BAE

	Values (n, %)		***P***
	Onset of endophthalmitis	Final follow-up	
VA Snellen			
20/400 or better	1 (3.6%)	8 (28.6%)	
CF	1 (3.6%)	1 (3.6%)	
HM	9 (32.2%)	8 (28.6%)	
LP	14 (50.0%)	6 (21.4%)	
NLP	3 (10.7%)	5 (17.9%)	
LogMAR	2.58 ± 0.27	2.14 ± 0.85	≤ 0.001
IOP (mmHg)			0.186
Mean ± SD	18.7 ± 13.8	14.9 ± 6.0	
Range	3–60	7–37	
The number of patients with IOP ≥ 30, n (%)	5 (17.9%)	1 (3.6%)	
The number of patients with IOP ≤ 6, n (%)	1 (3.6%)	0 (0%)	
The number of patients of normal IOP without surgery, n (%)	16 (57.1%)	22 (78.6%)	
Further surgery for IOP control		5 (17.9%)	
Ahmed glaucoma valve implantation		3 (10.7%)	
Ex-PRESS implantation		1 (3.6%)	
Cyclophotocoagulation		1 (3.6%)	
Enucleation		2 (7.1%)^a^	

*VA* Visual acuity, *BAE* Bleb-associated endophthalmitis, *CF* Counting fingers, *HM* Hand movement, *LP* Light perception, *NLP* No LP

^a^One eye with BAE, one with absolute glaucoma

In this study, we attempted to preserve visual function and reduce the rate of blindness. Visual acuity was poor in most individuals at the onset of BAE, as only one eye (3.6%) had a VA of 20/400 or better. Fortunately, the visual acuity were significantly improved from 2.58 ± 0.27 to 2.14 ± 0.85 (reported in logMAR) after treatment (*p* ≤ 0.001), and the cases presented with VA of 20/400 or better raised from 3.6% to 28.6% after reasonable treatment. The eyes that presented with VA of light perception reduced from 14 (50.0%) to 6 (21.4%) after therapy. However, two other patients ended up in NLP at the final follow-up. Two patients underwent enucleation at the final follow-up: one eye with BAE and the other with absolute glaucoma. One eye, whose culture-proven causative microorganism was *C. albicans* (fungi), underwent PPV three times (twice in another hospital and once in our hospital), and enucleation was performed 6 months after discharge because of eyeball atrophy. Another eye, which was *Staphylococcus hominis* culture-positive, underwent PPV once but ended with enucleation because of absolute glaucoma 2 years after discharge.

The mean IOP was 18.7 ± 13.8 mmHg (range = 3–60 mmHg) at the time of diagnosis of BAE and 14.9 ± 6.0 mmHg (range = 7–37 mmHg) at the final follow-up. A large proportion of patients (78.6%) achieved normal intraocular pressure without further glaucoma surgical techniques at the final visit. Four patients underwent glaucoma filtration surgery after discharge. Three patients underwent Ahmed glaucoma valve implantation, and one underwent Ex-PRESS implantation and had a better IOP value. Only one patient who recently underwent cyclophotocoagulation still had an uncontrolled IOP of 37 mmHg at the final follow-up and required close observation.

### Comparison between two periods

Given the challenges in accessing medical treatment during the COVID-19 pandemic in China these past 3 years, we compared the changes in BAE during the COVID-19 pandemic (from 2020–2022) and previous years (from 2012–2019) in our hospital.

**Fig. 1:**
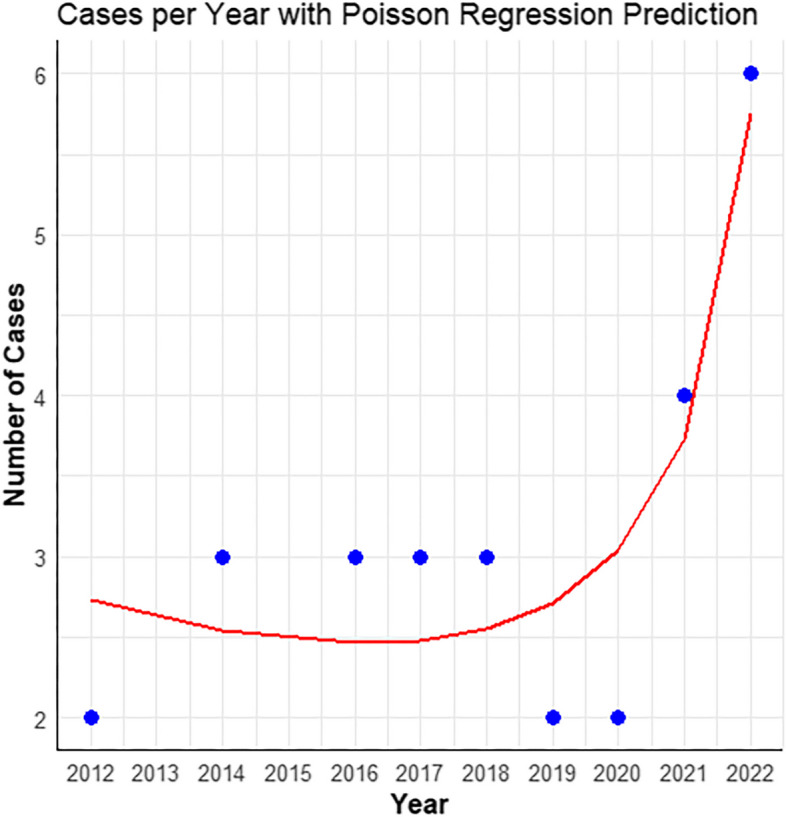
Poisson regression model analysis showed a significant upward trend during the COVID-19 pandemic period from 2020–2022, compared to previous years. The predictions made by the Poisson regression model closely aligned with the actual data, especially in depicting the general trend of case number changes before and during the pandemic

These characteristics (age, sex, bleb condition, organism, treatment process, IOP, and visual acuity at the onset of BAE and final follow-up) are listed in Table 6. A total of 12 patients acquired BAE during the COVID-19 pandemic, with an average of 4 cases per year. A total of 16 cases were reported in previous years, with an average of 2 cases per year. The annual incidence of BAE during the COVID-19 pandemic period was twice greater than that of previous years. Furtherly, we found a significant increase in case numbers during the COVID-19 pandemic period from 2020 to 2022, which showed a marked upward trend compared to previous years via Poisson regression model analysis (Fig. 1). Additionally, the predictions made by the Poisson regression model closely aligned with the actual data, especially in depicting the general trend of case number changes before and during the pandemic. The fitted equation is: Cases = 233.865 − 0.115 × Year − 0.789 × Offset. This equation represented the relationship between the number of cases (Cases) and the year (Year), as well as the offset (Offset). The coefficient for the year was -0.115, which indicated a slight downward trend in case numbers as the year increased, while the coefficient for the offset is -0.789, which furtherly adjusted the impact of this trend.

**Table 6:** Comparison between two periods

	BAE presented years		***P***
	2020–2022 (*n* = 12)	2012–2019 (*n* = 16)	
Age, mean ± SD, y	45.2 ± 14.5	52.8 ± 14.1	0.155
Male, n (%)	10 (83.3%)	10 (62.5%)	0.403
Interval between glaucoma surgery and BAE, mean ± SD, y	10.8 ± 5.5 (rage 4.0–20.0)	9.1 ± 5.3 (rage 0.2–19.0)	0.403
Interval between symptoms and treatment, mean ± SD, d	5.8 ± 9.6 (rage 0.42–60.0)	4.4 ± 4.7 (rage 1.0–20.0)	0.092
IOP at onset of BAE, mean ± SD, mmHg	22.9 ± 15.4 (rage 9.6–60.0)	15.4 ± 15.0 (rage 3.0–52.0)	0.163
IOP at the final follow-up, mean ± SD, mmHg	16.7 ± 8.1 (rage 7.0–37.0)	13.6 ± 8.4 (rage 7.0–19.0)	0.192
Presence of a bleb leak, n (%)	4 (33.3%)	5 (31.3%)	1.000
PPV combined with tap-and-inject			1.000
Initial tap-and-inject	5 (41.7%)	6 (37.5%)	
Initial PPV	7 (58.3%)	10 (62.5)	
Number of tap and inject	1.4 ± 0.7	1.3 ± 0.7	0.513
Number of PPV	1.3 ± 0.7	1.1 ± 0.3	0.424
Organisms culture-positive, n (%)	8 (66.7%)	7 (43.8%)	0.229
VA at onset of Endophthalmitis (logMAR)	2.5 ± 0.2	2.6 ± 0.3	0.642
VA at final follow-up (logMAR)	2.0 ± 0.9	2.3 ± 0.8	0.372

*VA* Visual acuity, *BAE* Bleb-associated endophthalmitis, *CF* Counting fingers, *HM* Hand movement, *LP* Light perception, *NLP* No LP

We found that male patients accounted for an overwhelming percentage of bleb-associated endophthalmitis during the two periods:10 (83.3%) patients in the COVID-19 period and 10 (62.5%) patients in the previous period. The proportions of bleb leaks and treatment processes were similar in both periods. The interval between symptoms and treatment was similar in the COVID-19 pandemic (5.8 ± 9.6 days) periods and previous years (4.4 ± 4.7 days). The ratio of visual acuity better than 20/400 at final follow-up was seemingly higher during the COVID-19 pandemic period (5 cases, 45.7%) than previous years (3 cases, 18.8%) with no significance.

## Discussion

In our study, we found that POAG filtering surgery, male sex and the COVID-19 pandemic period might be potential interrelated factors for BAE. *Streptococcus* species were the leading pathogenic organisms isolated in the current research.

IOP is one of the most important risk factors for glaucoma [13, 14], and glaucoma filtration surgery is an effective approach to decreasing it [2]. The presence of a filtering bleb after the surgery may result in bleb-associated endophthalmitis, which is rare and could be a devasting complication [8, 15, 16].

From the patient demographic investigation in our study, we found that the proportion of BAE was approximately 0.3%, which is close to other reported studies [9, 11, 17–20], varying from 0.2% to 3.8%. Also, we found most of them were male patients (71.4%, *p* = 0.023), which was consistent with the reports that male sex might be a potential correlative factor for BAE [21, 22].

Consistent with our previous study on BAE duration from 2003–2010 [8], POAG in the current study (39.3%, *p* ≤ 0.001) was the most common glaucoma related to BAE. Although a national cross-sectional study has shown that PACG accounts for the largest proportion in China [23], POAG has been considered a relative factor in bleb-related infections [6, 8]. The reasons can be considered from two perspectives. On the one hand, patients with POAG are younger and more prone to scarring than those with PACG, leading to longer use of the subconjunctival infiltration of antimetabolites to achieve a lower IOP, potentially predisposing to bleb-related infection [6, 22]. On the other hand, the surgical options for PACG have changed in recent years, and an increasing number of doctors are inclined to select cataract extraction combined with goniosynechialysis instead of traditional glaucoma filtration surgery [10].

Gathering statistics on antimetabolite usage was challenging in this study, as most patients had previously undergone glaucoma filtration surgery in other primary hospitals, except for nine patients who had previously undergone glaucoma surgery in our hospital and who received MMC adjunctive infiltration. Previous research has confirmed that antimetabolites are a risk factor for BAE and can induce thinner and more avascular filtering blebs. Thin-walled and avascular blebs were considered more inclined to become infected than thicker blebs [5, 20, 22, 24]. Patients with BAE visited our clinic with bleb infection and pus in the bleb, as well as thin and avascular blebs, suggesting that the bleb condition might be one of the related factors involved in the pathogenesis of BAE. In addition, bleb leaks detected in 9 patients (32.2%) in our current study and in other studies were also regarded as potential related factors for BAE. Bleb leaks might lead to hypotony and a vulnerable ocular surface, which is the barrier preventing invasion from bacteria and contributes to blebitis or endophthalmitis [9, 19].

All the cases in our study had late endophthalmitis, with an average time of approximately 9.8 years from glaucoma filtration surgery, longer than in some studies [9, 25]. In accordance with other studies, most of the patients visited the hospital with complaints of redness, reduction of vision, and ocular pain [8, 9, 24]. As most patients had endophthalmitis many years after the glaucoma filtration process, they should be advised by ophthalmologists about the significance of redness, blurred vision, and ocular pain as symptoms, and encouraged to promptly seek medical attention.

Visual acuity was one of the most important assessment criteria for ocular disease severity and treatment outcomes. In our study, only one patient had a VA better than 20/400 at the onset of BAE. Consistent with other reports, most patients diagnosed with BEA had poor initial visual acuity before treatment [8, 9, 24, 26], which might result from corneal edema and an inflammatory response in the vitreous humor. Fortunately, the visual acuity was significantly improved after treatment (*p*≤0.001), which reminded us of the importance of timely treatment in rescuing the vision of patients with BAE.

The abnormal intraocular pressure at the onset of BAE observed in the present study is noteworthy. Seven (25.0%) eyes showed a low tension of < 10 mmHg), and one patient presented with hypotony < 6 mmHg, which might be related to poor bleb conditions like bleb leak and impaired ocular surface. The fragile surface surroundings provided a path for the invasion of pathogenic microorganisms, resulting in blebitis or endophthalmitis [9, 19]. Meanwhile, we noticed that five patients (17.9%) exhibited abnormally high IOP > 30 mmHg, with the highest IOP value being 60 mmHg. Quite a few studies account for this phenomenon. Sacc`a et al. indicated that inflammation in the anterior segment might elevate aqueous outflow resistance, promote trabecular meshwork dysfunction, and increase IOP in POAG [27]. In our study, we hypothesized that the inflammatory response in the anterior chamber and vitreous cavity may result in filtration outflow channel obstruction or dysfunction and contribute to ocular hypertension at the onset of BAE.

In our study, all patients received PPV combined with the tap-and-inject procedure, most patients (85.7%, < 0.001) underwent single PPV. However, some underwent PPV or tap-and-inject more than once for inflammatory inhibition. Some studies have shown that PPV for BAE results in improved visual outcomes [26, 28–30]. However, Islam et al. confirmed that visual acuity and IOP control were similar after initial PPV compared with taps and injections when managing BAE [31]. Leng et al. found that patients with BAE had better visual acuity via tap and injection than patients with PPV [18].

During surgery, specimens were acquired from the aqueous and vitreous chambers to detect the causative infectious agents. *Streptococcus spp*. were the most common pathogenic organisms found in our study (46.4%, *p* ≤ 0.001). Similar findings have been reported in some research [9, 11, 25, 26, 29]. Our previous study and some researchers noted that *Staphylococcus* species were more commonly observed [8, 32]. Although microbiological cultivation was affected by technical means, the microbial spectrums for BAE have not changed much over the years, and gram-positive bacteria are still the most frequent organisms.

At the final follow-up, the visual acuity was significantly increased after treatment (*p* ≤ 0.001), and the cases presented with VA of 20/400 or better were raised from 3.6% to 28.6% after reasonable treatment. This indicates that timely treatment may improve visual acuity in some patients. However, patients with NLP raised from 3 to 5 cases at the final follow-up, and even ended up with enucleation because of eyeball atrophy or absolute glaucoma. Busbee and Jacobs et al. confirmed that culture-positive cases were related to worse VA outcomes than culture-negative cases [26, 33]. In accordance with previous research, both eyes in our study that ended with enucleation were culture-positive, indicating that organisms affected visual outcomes. Most of the cases (78.6%) in our study acquired a stable IOP after surgery at the final follow-up. However, four patients accepted further glaucoma surgeries and had stable IOP at the final follow-up. Another BAE patient, who accepted cyclophotocoagulation in 2022, still had ocular hypertension at the final follow-up. The proportion of stable IOP control was higher than that of Mady et al., who found that 25% of patients with BAE could maintain stable IOP control after treatment [34].

The COVID-19 pandemic has made it difficult for people to receive medical services throughout China. In this study, we compared the clinical processes between the COVID-19 pandemic and previous years. The cases of BAE occurring annually during the COVID-19 pandemic period (4 cases per year on average) were twice as many as in the previous years (2 cases per year on average). Poisson regression model analysis showed a significant upward trend during the COVID-19 pandemic period from 2020–2022, compared to previous years, which indicated the COVID-19 pandemic might be a potential related factor for increased incidence rate of BAE. We noticed that, with non-statistical meaning, the proportion of patients with VA 20/400 or better at the final follow-up was seemingly higher during the COVID-19 pandemic period. Several hypotheses can explain this phenomenon. On the one hand, researchers put forward the idea that a digital revolution was needed to face the COVID-19 pandemic [35], and ophthalmologists have been striving to provide medical care most effectively via telemedicine during the COVID-19 pandemic [36]. Consistent with other reports, doctors in our hospital struggled to provide reliable and timely advice via online outpatient services. However, patients might pay sufficient attention to their self-health status and eye discomfort during the COVID-19 pandemic and visit doctors promptly.

As this was a descriptive report with only 28 cases with BAE included from 2012–2022, our data analysis and hypothesis were restricted by the number of cases in the current study. Further research on BAE is required in our future studies, and the time range of the data search should be expanded to make our results more significant.

## Conclusion

To summarize, BAE remains an intractable disease that can result in irreversible visual impairment and even loss of the eyeball. POAG with filtration surgery was the most common type of glaucoma associated with BAE, the COVID-19 pandemic period and male sex may be a potentially relevant factor for BAE. Culture positivity is closely related to BAE prognosis, and *Streptococcus spp.* are the leading pathogenic organisms in BAE. Emphasizing the educational management of patients after glaucoma filtering surgery is necessary. Online outpatient services, early diagnosis, and timely treatment may rescue vision and maintain intraocular pressure control when BAE is present.

## Data Availability

The data presented in this study are available on request from the corresponding author.
